# Ultrasound Communications Reveal Social Aversion in Rats With Contact Motivation Deficits but not Anhedonia

**DOI:** 10.31083/AP43990

**Published:** 2025-06-18

**Authors:** Anastasiya A. Rebik, Nadezda D. Broshevitskaya, Vyacheslav D. Riga, Pavel L. Aleksandrov, Maria I. Zaichenko, Inna S. Midzyanovskaya

**Affiliations:** ^1^Institute of Higher Nervous Activity and Neurophysiology of the Russian Academy of Sciences, Laboratory of Human Higher Nervous Activity, 117865 Moscow, Russia; ^2^Institute of Higher Nervous Activity and Neurophysiology of the Russian Academy of Sciences, Laboratory of Neurophysiology of Emotions, 117865 Moscow, Russia; ^3^Neuroscience Department, Sirius University of Science and Technology, 354340 Sochi, Russia; ^4^Institute of Higher Nervous Activity and Neurophysiology of the Russian Academy of Sciences, Laboratory of NeuroOntogeny, 117865 Moscow, Russia

**Keywords:** animal model, autism spectrum disorder, behavior, emotion, social contact deficit, ultrasound communication

## Abstract

**Objectives:**

Animal models of human diseases are commonly used in experimental research. Autism Spectrum Disorder (ASD) is characterized by reduced social motivation and often co-occurs with epilepsy, representing a distinct clinical subgroup. This study investigated social deficits in Krushinsky-Molodkina (KM) rats, which present with latent epilepsy and an autistic-like phenotype, by measuring ultrasonic communications during social interaction.

**Methods:**

The three-chamber test for social preference/novelty, accompanied by registration of ultrasonic vocalizations (USVs), was conducted in 12 KM and 12 control Wistar rats. For analysis, each individual vocalization trajectory was mapped and the results were derived from aggregating the individual data. To assess potential anhedonia, sucrose preference was tested in a separate group (10 KM and 20 Wistar rats) by measuring the consumption of 1% sucrose and water in individual rats over a 24-h period. All animals used were seizure-naive males, aged 4–6 months.

**Results:**

A longer duration of aversive USV calls was registered during the sociability tests in KM rats (*p* < 0.05, compared with controls). The majority (*p* < 0.05) of aversive USVs occurred when KM rats distanced themselves from the social stimuli, and the duration of these calls showed a positive correlation with freezing behavior (Spearman coefficient Rs = 0.68, *p* < 0.05). Reduced sucrose preference was not observed in KM rats; instead, an increase in daytime sucrose consumption was noted.

**Conclusions:**

KM rats exhibit negative emotional states in sociability tests, as evidenced by enhanced aversive vocalizations and distancing behavior. The social aversion observed in KM rats is not associated with anhedonia.

## Main points

∙ KM rats show social aversion, marked by distancing behavior and aversive ultrasonic vocalization.

∙ The negative emotional state in KM rats is not associated with anhedonia.

∙ The KM rat strain is a potential animal model for comorbid epilepsy and autism spectrum disorder.

## 1. Introduction

Social deficits are a characteristic symptom associated with a wide range of neurological and psychophysiological disorders, including schizophrenia, depression, autism spectrum disorder (ASD), and organic brain damage [1, 2, 3, 4, 5]. Social deficits are characterized by reluctance or inability to engage in social interactions with both familiar and unfamiliar conspecifics. This contact motivation deficit is most pronounced in individuals with ASD. ASD is diagnosed in 2%–3% of children, making it one of the most prevalent developmental disorders [6].

The heterogeneity of behavioral manifestations of ASD and comorbid disorders underscores the need for personalized clinical approaches, including rational clustering of patient cohorts. Recent reviews report that the prevalence of epilepsy in individuals with ASD is 13% (range 2%–60%) and the prevalence of autism in epileptic patients is 9% (range 1%–42%) [7, 8]. Thus, the subset of patients with comorbid epilepsy (also latent epilepsy) and ASD represents a unique target for translational research.

Prenatal and postnatal valproate administration in laboratory rodents is the most commonly used approach to generate animal models of ASD comorbid with attention deficit hyperactivity disorder (ADHD), as it reproduces the specific ASD-ADHD phenotype [9, 10, 11, 12]. A limitation of this model is the need for invasive procedures, lowered pup survival rates, and the potential co-occurrence of other developmental abnormalities. It has previously been demonstrated that Krushinsky-Molodkina (KM) rats exhibit persistent deficits in social interactions with healthy conspecifics [13, 14]. Consequently, this strain could serve as a potential model for ASD. The KM strain is inbred and genetically predisposed to audiogenic seizures, making it one of the oldest animal models of human convulsive epilepsies [15]. The reflex type of audiogenic seizures in KM rats allows researchers to control the seizure experience in a non-invasive manner. Although the pathophysiology underlying social deficits in KM rats remains poorly understood due to the novelty of the approach, preliminary insights have been proposed. A recent transcriptome study in KM rats reported overexpression of the interleukin-I receptor and cytokines vulnerability, suggesting an increased vulnerability to neuroinflammation [16]. Neuroinflammation has long been implicated as a potential causal factor for ASD symptoms [17, 18, 19]. Furthermore, dysfunction of the insular cortex—a brain region relevant to autism pathobiology—has been observed in KM rats, in terms of imbalanced binding to D1-like and D2-like dopamine receptors [20, 21, 22, 23].

Communication deficits are a core diagnostic criterion for ASD [6, 24]. Although this phenomenon is challenging to directly translate into animal models, substantial research efforts have been directed towards this area.

Animal communications can involve various modalities, including tactile (e.g., touches and mutual grooming), olfactory (e.g., sniffing and following odor traces), and auditory signals [25, 26]. The present study focuses on the ultrasonic communication between conspecifics, which is observed in sociability tests.

In juvenile and adult rats, two primary types of ultrasonic vocalization (USV) are observed, depending largely on the emotional valence of the context. In situations with a negative emotional load, such as inter-male aggression, predator exposure, and fear learning paradigms, rats emit highly stereotyped aversive USV [27, 28, 29, 30, 31]. Aversive USVs (near 22 kHz) inform conspecifics of the negative affective state of the sender, and of a possible external threat [32]. The playback of aversive calls is known to produce in rats a response similar to those evoked in human brains by the presentation of fearful faces, activating brain regions associated with negative valence systems [32].

**Fig. 1.**
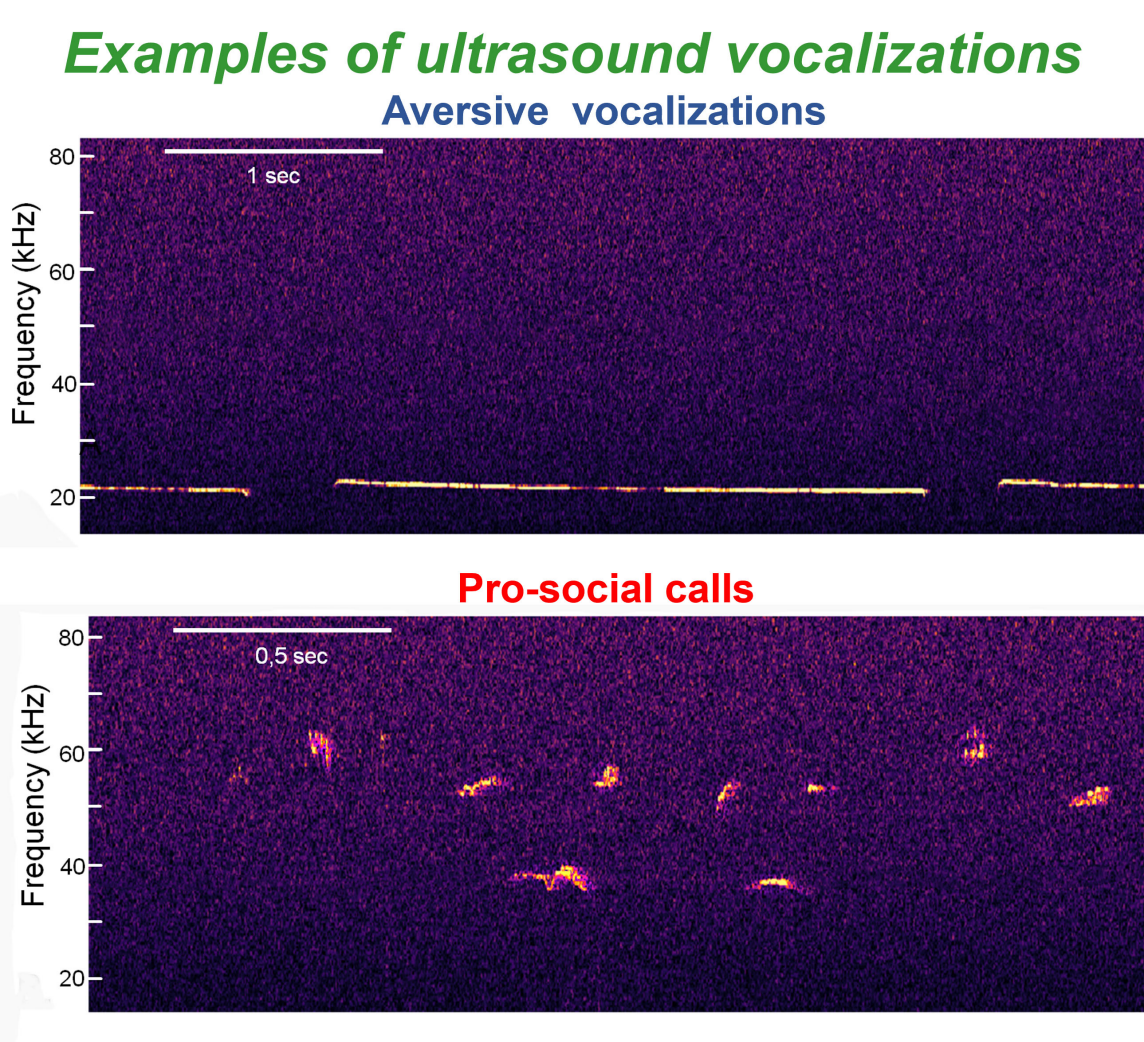
**Examples of aversive (upper panel) and pro-social (lower panel) ultrasound vocalizations observed during the social preference tests (see also Results Section)**.

These calls are confined to a relatively narrow peak frequency range of 18 to 24 kHz. Compared with other USV types, aversive USVs exhibit low variability in most acoustic features, making them relatively conserved. Examples are shown in Fig. 1, upper panel. In contrast, other types of USVs consist of short, variously shaped events, with a frequency range of 35–80 kHz (Fig. 1, lower panel). These are typically described as pro-social calls, as they promote social approach, play behavior, and food-seeking activities [27, 33, 34, 35].

The primary aim of this study was to assess the emotional valence of social loads in two cohorts of animals, differing in their sociability traits, by analyzing the characteristics of USVs. Additionally, we conducted an initial contextual analysis through virtual compartmentalization of ultrasound and locomotor activity.

## 2. Methods

### 2.1 Animals

The experiments were conducted on adult males of KM and Wistar strains (n = 22 and n = 32, correspondingly), aged from 4 to 6 months, and weighing 250–400 g. KM rats were provided by the exclusive breeder, the Biological Faculty of Moscow State University, and Wistar rats were obtained from the Stolbovaya animal supplier. All animals were kept in standard plastic cages (L × W × H, 53 × 32 × 17 cm), in groups of four to six rats, under a 12-h light/dark cycle, with food and water ad lib. All the animals arrived at the Animal Chapter of Institute of Higher Nervous Activity and Neurophysiology of the Russian Academy of Sciences (IHNA) at the age of 90–120 days. In the current study, as well in previous ones, Wistar rats were used as healthy controls, as they are the founding strain of KM rats [13, 14, 23].

### 2.2 Ethical Note

All experiments were conducted in accordance with Directive 2010/63/EU on the protection of animals used for scientific purposes. The study was approved by the Institutional Ethics Committee, and all procedures followed the principles outlined in the Animal Research: Reporting of In Vivo Experiments (ARRIVE) guidelines. Every effort was made to minimize the unnecessary discomfort of the experimental animals.

### 2.3 Social Preference/Social Novelty Tests

**Fig. 2.**
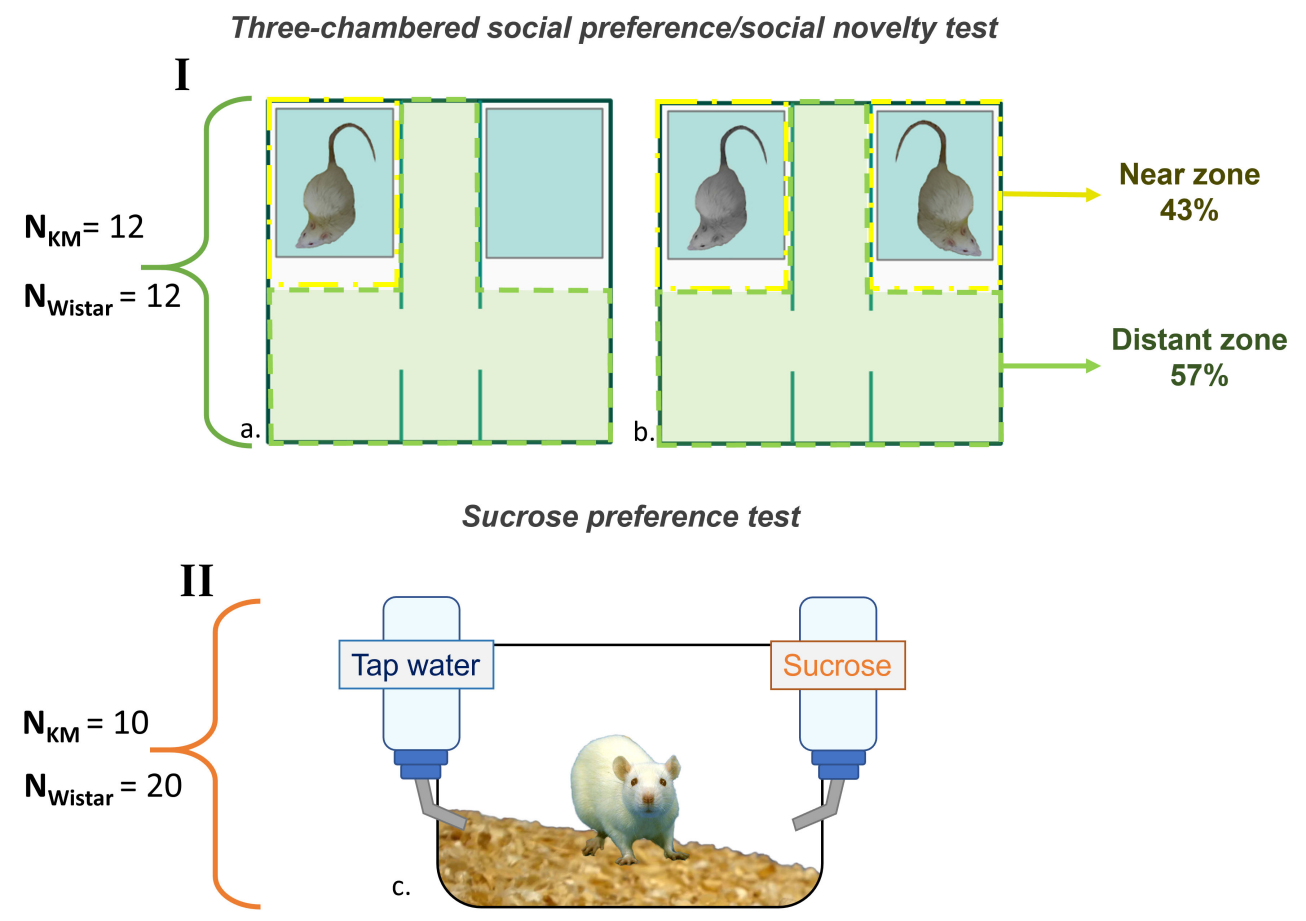
**The schematic of behavioral experiments**. (a) The social preference/social novelty test. (b) The social preference test. (c) The sucrose preference test. For each experimental cohort, the corresponding strain sample sizes shown given on the left. KM, Krushinsky-Molodkina. The picture was made by us (A.Rebik), using Microsoft PowerPoint 2021 (Microsoft, Redmond, WA, USA) and Inkscape 1.4 (licensed under GNU GPL V2, Boston, MA, USA). We photographed the rats for the picture.

The sociability tests were originally introduced for voles and mice [36, 37, 38, 39]. We used modified versions of these tests, as described in previous studies [13, 14]. Modifications included changes to the apparatus material, as well as adjustments to the arena size and the shape of the social stimuli containers. The three-chamber apparatus consisted of a 60 × 60 cm black arena with 60-cm high black wooden walls on the perimeter and two nontransparent walls inside, dividing the arena into two chambers and a central rectangular compartment (Fig. 2). Each divider had an open entrance to allow free passage between the compartments. Each chamber contained a dark perforated plastic box (15 × 20 × 15 cm): one box housed an unfamiliar male rat, while the other contained only bedding from the home cages of social stimulus animals. Social stimuli were unfamiliar Wistar rats of similar age and weight. For each trial, they were chosen from a single cage in the same animal facility as the experimental animals. Therefore, the social stimulus boxes were as similar as possible in all aspects, including olfactory cues.

In the social preference test, the setup included an empty box and a box containing a stranger rat (Fig. 2a). For the social novelty test, the empty box was replaced with an identical container housing a new unfamiliar rat, which was a cage mate of the first stranger rat used in the social preference session (Fig. 2b). The experimental schedule involved alternating the left and right compartments for different test animals, in order to balance potential external influences. Each test session lasted 10 min, with behavioral observations recorded throughout. The arena was cleaned and wiped between the end of the social novelty test session for one rat and the beginning of the social preference test session for the next animal, to minimize potential olfactory cues. Before the tests, the home cages of the experimental animals were brought to the experimental room, where they were allowed to acclimate to the environment for 1 h. During this time, the social stimuli were placed in their respective boxes (as described above), each containing bedding from their home cages. The third identical box remained empty, with two handfuls of bedding from the home cages placed inside. To habituate to the arena, the test animals were allowed to explore the environment for 10 min in groups with their cage mates. Immediately following the habituation period, the social preference/novelty tests were conducted.

### 2.4 Sucrose Preference Test

The sucrose preference test was conducted over 2 days with individual KM and Wistar rats [40]. Each rat was placed in a separate plastic cage, containing two bottles: one with 1% sucrose solution and the other with tap water (Fig. 2c). No food or water deprivation was implemented. The bottles were weighed, and their positions alternated every 12 h. The first day of the test was considered as a habituation period. Sucrose and water consumption was calculated for each rat, for the second day of the test.

### 2.5 Offline Tracking and Behavioral Observations 

Video recordings of the sociability tests were processed using the free offline video tracker ToxTrac (version 2.98, Umea University, Umea, Sweden) [41]. The software was employed to calculate the trajectory and total distance traveled, the time spent in the selected compartments, and the number and duration of freezing episodes. Additional visual analysis of behavioral events was conducted offline by an independent, blinded expert. Rearings, full-body (long) and facial (short) grooming, and animal-to-animal contact were evaluated. Animal-to-animal contact was defined as the test animal’s snout poking at the containers housing the unfamiliar rats.

### 2.6 Ultrasound Detection and Mapping

Along with the sociability tests, ultrasonic vocalizations were recorded using the ultrasonic recorder ‘Sonotrack’ (Metris; Hoofddorp, the Netherlands), with the microphone positioned 1 m above the test chamber floor. Ultrasound was registered throughout the entire experimental sessions (each lasting 10 min). Offline analysis was performed automatically by the DeepSqueak neural network based on MatLab ( https://github.com/DrCoffey/DeepSqueak), followed by manual expert verification [42]. The number and duration of aversive calls (19–33 kHz, from 0.5 to 5 s) were extracted, as well as the parameters of pro-social vocalizations (40–60 kHz, up to 1 s). For the zonal analysis of USVs, the arena was divided into two zones: the near zone (i.e., the vicinity of the guest rats’ containers) and the distant zone (stimulus-free parts of the chambers and the corridor between the chambers; Fig. 2a,b). Each detected USV was assigned to the corresponding track points, and then the points were categorized according to the geometry of the two zones (Fig. 2a,b). For statistical analysis, the numbers and duration of USVs in each zone were examined.

The animal tracks were combined by superimposing the individual tracks onto one another. To create a clear overlay, each image was made 25% transparent. All images were oriented so that the first stimulus rat was positioned in the upper left corner. The images were generated using the SciPy library in Python (SciPy Version 15.3, Python version 3.12.1, https://scipy.org/). Vocalization episodes were labeled by identifying the start time of each USV relative to the animal’s track. Pin marks for aversive (blue) and pro-social (red) calls were placed at the time-specific coordinates of the individual tracks.

### 2.7 Statistics

For the sociability tests, the significance of the between-group factor (strain) was assessed using a two-tailed Kruskal-Wallis analysis of variance (ANOVA) by Ranks; all within-group effects were analyzed using the Wilcoxon matched-pairs signed-rank test. For the sucrose preference test, repeated measures ANOVA, with ‘strain’ as a between factor and ‘measurement’ as a within factor, was used. All within-group effects were analyzed using the Wilcoxon matched pairs test. Correlations were assessed using Spearman’s rank-order correlation method.

## 3. Results

**Fig. 3.**
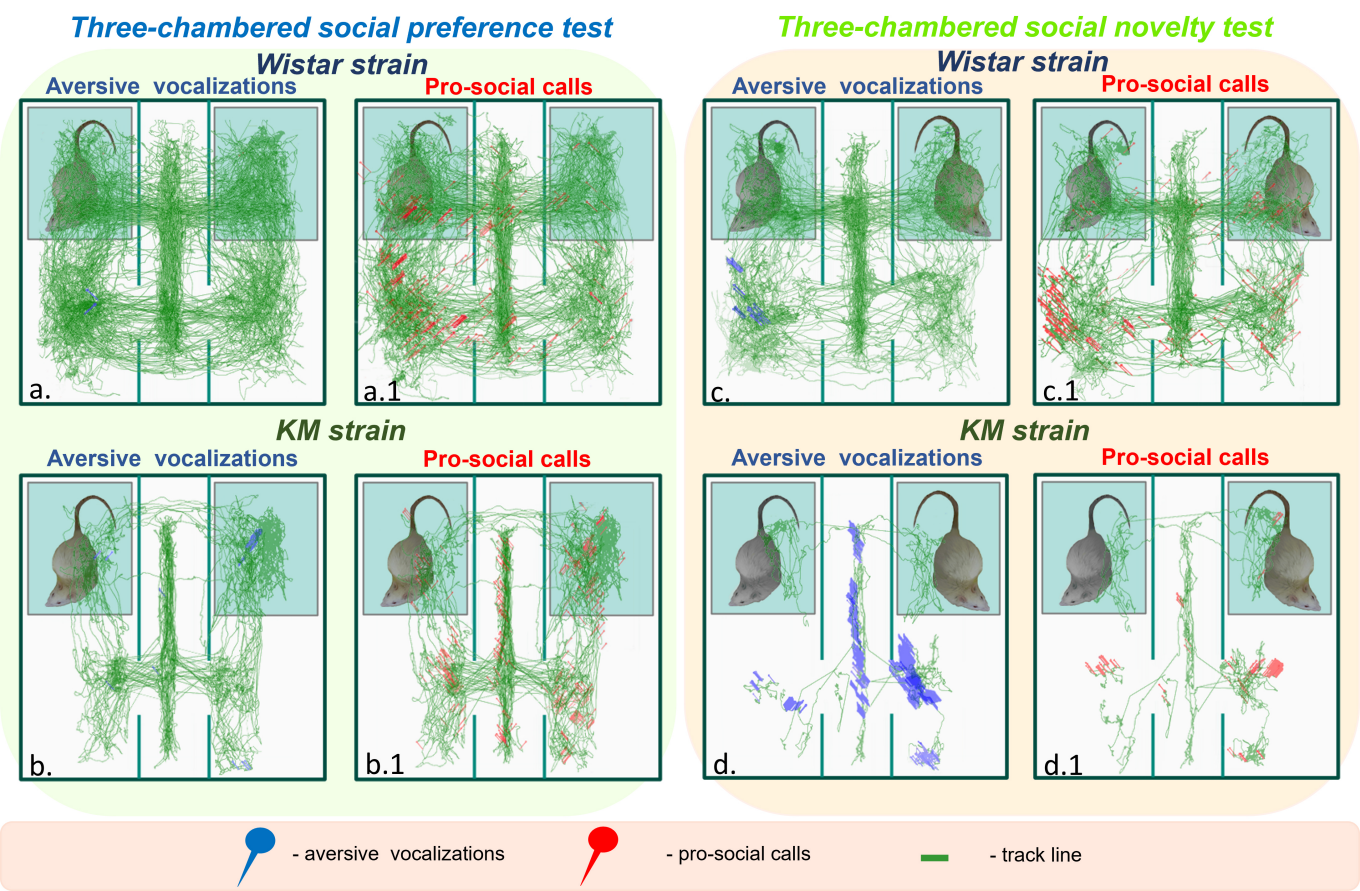
**The locomotor and vocal behaviors of Wistar and KM rats**. The green lines represent graphical summaries of all individual locomotor trajectories for each strain: Wistar rats are shown in the upper panels, while KM rats are shown in the lower panels. Each red or blue pin corresponds to a registered USV event: blue pins indicate aversive USVs and red pins represent pro-social USVs. Two sessions of the sociability tests are shown: the left panels (a,a.1,b,b.1), shaded in green, correspond to the social preference test, while the right panels (c,c.1,d,d.1), shaded in orange, correspond to the social novelty test. USV, ultrasonic vocalization.

**Fig. 4.**
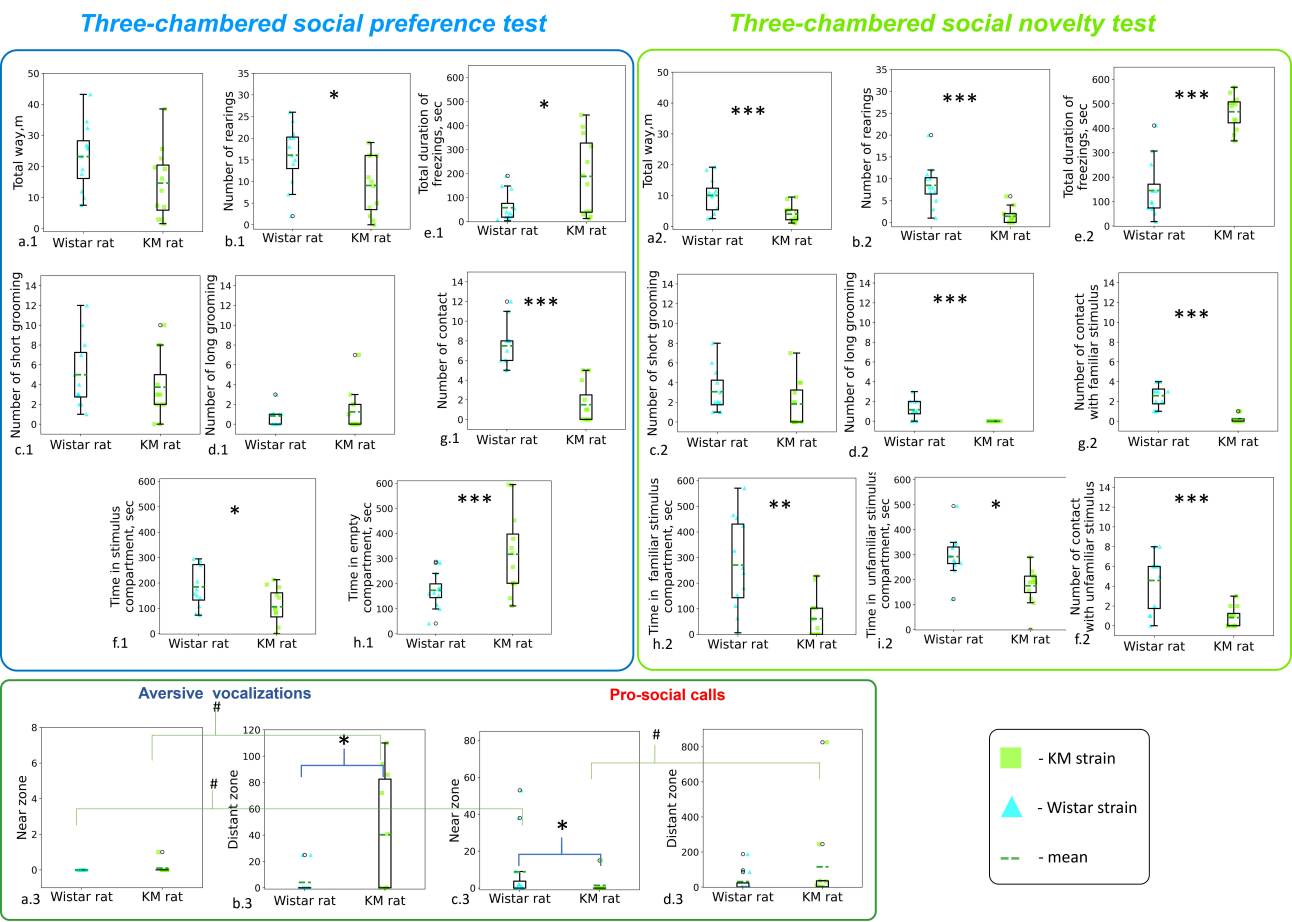
**Quantitative analysis of locomotor and vocal behaviors in Wistar and KM rats during the sociability tests**. (a.1–h.1) Locomotor behaviors in the social preference test; (a.2–i.2) locomotor behaviors in the social novelty test. (a.3–d.3) USV production during the social novelty test. Statistical significance is indicated as follows: Kruskal-Wallis ANOVA by Ranks ^*⁣**^p < 0.001, ^**^p < 0.01, ^*^p < 0.05; Wilcoxon matched pairs test, ^#^*p* < 0.05.

The majority of sociability tests were accompanied by ultrasound emission, with the exception of two test sessions in the Wistar cohort. Ultrasound events were detected in overlapping instances in only 0%–3% cases, as cumulative vocalizations were recorded during these sociability experiments. Consequently, the emitted USVs originated both from the freely moving test rats and the social stimuli. Specifically, (1) in the social preference test (Fig. 2a; Fig. 3a,a.1,b,b.1), and (2) in the social novelty test session (Fig. 2b; Fig. 3c,c.1,d,d.1). Locomotor and contact behaviors were extracted from video recordings of the test rats only. The results (Fig. 4) revealed contact deficits in KM rats, which were associated with an emotionally negative valence of ultrasound rodent communications.

In the social preference test conducted for KM rats, an increased emission of aversive ultrasound calls was observed. These increases were seen both in the total number (H(1,21) = 6.42, *p* = 0.011) and cumulative duration (H(1,21) = 6.42, *p* = 0.028) of aversive calls. The total number and cumulative duration of pro-social calls registered during the social preference session (Fig. 3a.1,b.1) did not differ significantly between the strains. However, control (Wistar) rats displayed a predominance of pro-social over aversive USVs (*p* = 0.009; Fig. 3a,a.1), a pattern not observed in KM rats (Fig. 3b,b.1), where no significant difference was found between the numbers of aversive and pro-social calls. Spatial zonality did not influence ultrasound production during the test session.

Behaviorally (Fig. 4a.1–h.1), the KM cohort exhibited a lower number of social contacts (H(1,24) = 16.77, *p* < 0.001; Fig. 4g.1), demonstrated reduced exploration activity (as indicated by a lower number of rearings; H(1,24) = 4.96, *p *= 0.026; Fig. 4b.1), and a pronounced freezing response (H(1,24) = 5.07, *p* = 0.024; Fig. 4e.1).

A significant negative correlation was observed between the number of aversive and pro-social calls in KM rats (Rs = –0,76, *p* < 0.05). Additionally, the cumulative duration of aversive calls was positively correlated with the freezing response in KM rats (Rs = 0.68, *p* < 0.05). These correlations were not significant for Wistar rats.

The second session, three-chamber social preference test further highlighted strain differences. Specifically, the total number (H(1,22) = 5.34, *p* = 0.021) and cumulative duration (H(1,22) = 5.33, *p* = 0.021) of aversive calls were elevated in the social novelty tests run in KM rats, as compared with Wistar rats. In KM rats, the number of aversive calls during the social novelty session tended to be increased, as compared with Wistar rats (*p* = 0.06). No differences in ultrasound production were observed in the Wistar cohort.

Behaviorally (Fig. 4a.2–i.2), the control rats (but not KM rats) exhibited more episodes of comfort (long) grooming (H(1,24) = 13.03, *p* = 0.0003; Fig. 4d.2) and less freezing (H(1,24) = 15.87, *p* = 0.001; Fig. 4e.2). Control rats developed more contacts both with familiar (H(1,24) = 16.40, *p* = 0.0001; Fig. 4g.2) and unfamiliar (H(1,24) = 9.42, *p* = 0.002; Fig. 4f.2) social stimuli, and demonstrated higher exploration (seen as rearings, H(1,24) = 13.64, *p* = 0.0002; Fig. 4b.2) and locomotion (H(1,24) = 11.60, *p* = 0.0007; Fig. 4a.2), as compared with KM rats.

Spatial distribution of vocal activity was also different between the two strains. Specifically, tests with Wistar rats demonstrated a predominance of pro-social USVs over aversive USVs (*p* = 0.033), particularly in the near contact zone (*p* = 0.018; Fig. 4a.3,c.3). No distinct zonality was identified for the aversive calls recorded during the Wistar rat tests. The difference between the near and distant zones was not significant.

In contrast, increased vocal activity was registered when KM rats were in the distant zones (pro-social calls, *p* = 0.038, Fig. 4c.3,d.3; aversive USV calls, *p* = 0.046, Fig. 4a.3,b.3). Significantly fewer pro-social USVs were recorded when KM rats were in the near-contact zone, compared with control Wistar rats (*p* = 0.035; Fig. 4c.3). In addition, significantly more aversive USVs were detected when KM rats were in the distant zone (*p = *0.046; Fig. 4b.3). Spatial zonality also influenced the vocal behavior of control rodents. Specifically, Wistar rats emitted more pro-social than aversive USVs in the near zone (Fig. 4a.3,c.3) and showed a similar trend in the distant zone (Fig. 4b.3,d.3). This effect was not observed in KM rats.

Given the increased amount of aversive vocalization observed in the KM rat cohort (Fig. 4a.3,b.3), coupled with reduced locomotion (Fig. 4a.2), an additional test for behavioral depression was conducted. Rats from both strains were individually tested for 1% sucrose consumption (Table 1). The results showed significant effects of strain (F(1,28) = 9.9, *p* = 0.003) and measurement (F(1,3) = 177.9, *p* < 0.0001), as well as a significant interaction between the two factors (F(3,84) = 6.5, *p* = 0.0005). Closer analysis revealed no significant differences in liquid consumption during the night; however, KM rats exhibited increased 1% sucrose intake during the daytime (F(1,29) = 32.9, *p* < 0.0001).

**Table 1.** **Sucrose preference test**.

	Liquid consumed, Wistar rats, mL (mean ± SEM)	Liquid consumed, KM rats, mL (mean ± SEM)	*p*-value
Day, Water	2.1 ± 0.4	2.5 ± 0.2	0.5 (ns)
Night, Water	2.1 ± 0.5	1.4 ± 0.3	0.4 (ns)
Day, Sucrose	8.1 ± 1.2	20.1 ± 2.0	0.000004
Night, Sucrose	32.1 ± 2.6	36.4 ± 2.6	0.3 (ns)

Values are presented as mean ± standard error of mean (SEM). Liquid consumption in rats of two strains. The volumes of 1% sucrose solution and tap water consumed by each rat were recorded every 12 h, following 24 h of habituation to the cages. Strain names: KM rats. ns indicates no significant difference. *p*-values were calculated according to the repeated measures ANOVA.

## 4. Discussion

In clinical studies, impaired empathy is observed in a range of psychiatric conditions, including autism [43]. Subjective human emotions are challenging to model in preclinical animal studies. However, many psychiatric conditions should also be considered in the context of emotional and motivational dysfunctions. Sociability tests are a key translational tool for studying social withdrawal [12, 44, 45]. Despite their utility, the interpretation of locomotor parameters obtained in these tests remains somewhat controversial, highlighting the need for further exploration of additional behavioral parameters to more accurately infer the emotional states of the model animals [46]. Rodent ultrasound vocal communication conveys information about the emotional valence of contexts, enabling researchers to infer the emotional states of the senders of USVs [26, 27, 32, 33]. Notably, distress calls in KM rats were predominantly observed in the distant zones, rather than in close proximity to social stimuli (*p = *0.035; Fig. 4a.3,b.3). Thus, taking into account the spatial social withdrawal and longer aversive (‘22 kHz’) vocalizations in KM rats (Fig. 4a.3,b.3), one can hypothesize that the rats with social contact deficits experience fear or anxiety. The amount of pro-social calls in the near zone also differed between the two strains, with control rats emitting more pro-social USVs. This finding further supports the hypothesis that these calls play a role in social affiliations and suggests the presence of social affiliation dysfunctions in rodents exhibiting autistic-like traits [33, 47].

The negative emotional state, as indicated by the increased production of aversive USV calls and freezing responses, should not be referred to as potential behavioral depression. Specifically, our results show that KM rats consumed a higher amount of 1% sucrose compared with Wistar rats. This could suggest a lower level of anhedonia in KMs relative to controls. However, there are two potential biases to considering this interpretation. First, according to existing literature, individuals with ASD often display a strong preference for sweet, carbohydrate-rich foods [48, 49, 50]. Thus, the increased sucrose consumption in the KM group may reflect the same tendency, potentially masking signs of depression. In addition, there was an observed circadian strain difference in the liquid consumption; the comparable night liquid intake contrasted with the elevated day-time liquid consumption (Table 1). This might indicate a dysfunctional sleep-wake cycle in KM rats, pointing to a translational parallel with the sleep disturbances that are often seen in ASD patients [51, 52, 53]. Altogether, these unexpected findings complicate the interpretation of depressive-like traits in KM rats, based solely on the sucrose preference test, which is considered the ‘gold standard’. Additional behavioral tests, including assessments of motivation, and possibly antidepressant trials, are needed for a more accurate evaluation.

The present study highlights the need for more precise zonation in the commonly used three-chambered social novelty/social preference test. Our findings suggest that not only the chambers themselves, but also the specific zones within these chambers, are critical for accurately assessing behavioral responses. The proximity to social stimuli within different zones provides valuable information on both pro-social locomotor activity and vocal communication in the experimental rats.

It is important to note that USVs, registered during both sessions of the sociability test, reflect a summation of the vocal activities of all participating animals. In all the tests, the social stimuli were made by the Wistar strain, meaning that the superpositions of Wistar-KM and Wistar-Wistar vocal communications were analyzed. However, strain differences, related to the altered contact activity, were still apparent. Another limitation of the study was the use of only male rats. Female KM rats were not available from the breeder, which restricts the ability to design experiments involving both sexes. In addition, the tests for sociability and sucrose preference were conducted on different, non-overlapping animal cohorts, which introduces a probabilistic limitation in the generalization of the results.

## 5. Conclusions

The social load encountered during the sociability tests revealed contact motivation deficits seen in ‘autistic’ KM rats. These rats showed reduced locomotor activity, heightened freezing responses, and a pronounced elevation in ultrasonic vocalizations associated with emotional aversion. Together, these findings indicate that KM rats, with an autistic-like phenotype, experience negative emotional states during forced social loads. Importantly, this negative emotional response in KM rats appeared to be specific to social contexts, as no depressive-like behavior was observed in the sucrose preference tests.

The study contributes further to the face validity of the KM rat strain as a potential animal model for ASD.

## Data Availability

The corresponding datasets can be obtained from the corresponding author by a reasonable request or viewed via the following link: https://github.com/RebikAnastasiya/DataSet_For_-Social-Aversion-in-Rats-with-Contact-Motivation-Deficits-but-Not-Anhedonia.
